# Volunteering and Its Association with Depression, Loneliness, and Lifestyle of Older Adults: Insights from a Japanese Cross-Sectional Study

**DOI:** 10.3390/healthcare12212187

**Published:** 2024-11-03

**Authors:** Thomas Mayers, Sachie Eto, Naoki Maki, Akihiro Araki, Hitomi Matsuda

**Affiliations:** 1 Department of Health Services Research, Faculty of Medicine, University of Tsukuba, 1-1-1 Tennodai, Tsukuba 305-8575, Ibaraki, Japan; 2 Medical English Communications Center, Faculty of Medicine, University of Tsukuba, 1-1-1 Tennodai, Tsukuba 305-8575, Ibaraki, Japan; 3 Department of Nursing, Faculty of Health Science Technology, Bunkyo Gakuin University, 1-19-1 Mukogaoka, Bunkyo, Tokyo 113-8668, Japan; s-eto@bgu.ac.jp; 4 Faculty of Rehabilitation, R Professional University of Rehabilitation, 2-12-31 Kawaguchi, Tsuchiura 300-0032, Ibaraki, Japan; maki@a-ru.ac.jp; 5 Health and Nursing Administration, Oita University of Nursing and Health Sciences, 2944-9 Megusuno, Oita-City 870-1201, Oita, Japan; araki@oita-nhs.ac.jp; 6 Graduate School of Comprehensive Human Sciences, Department of Human Care Science, University of Tsukuba, 1-1-1 Tennodai, Tsukuba 305-8575, Ibaraki, Japan; matsuda.hitomi.fn@u.tsukuba.ac.jp

**Keywords:** volunteer, older adults, depression, loneliness and social isolation, social connection, social prescribing

## Abstract

**Background/Objectives:** Volunteering has been widely recognized as beneficial to the well-being of older adults, providing health benefits, increased social engagement, and a sense of purpose. This study aimed to explore the associations between volunteering and physical and mental health measures among older adults in Japan. **Methods:** Using an online survey conducted during the COVID-19 pandemic, 500 participants aged 65 and older were divided into volunteer and non-volunteer groups. Demographic, lifestyle, and health characteristics were comprehensively assessed using a series of reliable and validated instruments. Logistic regression analysis was used to explore the associations between volunteering and health outcomes. **Results:** The findings revealed that while there were no significant differences in physical health indicators and undertreatment of most diseases (with the exception of cataracts), volunteers reported significantly lower levels of depression and loneliness compared to non-volunteers. The volunteer group also showed greater engagement in social activities and hobbies, which may have contributed to their improved mental health outcomes. **Conclusions:** The results of this study add to the growing body of evidence suggesting that volunteering may be an effective, low-cost intervention for promoting mental health and social engagement among older adults.

## 1. Introduction

Volunteering has consistently been shown to offer numerous benefits, particularly for older adults [1,2,3]. Engaging in volunteer activities for older adults can provide not only a sense of purpose and fulfillment [4] but also opportunities for social interaction [5], personal growth [6], and improved well-being [1,2,3,4,5,6,7]. For older adults, the advantages of volunteering extend beyond emotional satisfaction and social connection: it can also play a vital role in maintaining physical and mental health [1,8]. A review by Anderson and colleagues, for example, suggested that volunteering among older adults was associated with reduced symptoms of depression, better self-reported health, fewer functional limitations, and lower mortality [1]. A large-scale longitudinal study from Japan showed that engagement in voluntary work was protective against increased levels of depression among older adults [9].

In Japan, the aging population is one of the most pressing social challenges, with nearly 30% of the population aged 65 or older, which has important implications for Japanese healthcare and particularly the long-term care insurance system [10,11]. The rapid demographic shift, coupled with changes in traditional family structures, has led to increased concerns about loneliness, social isolation, health, and the overall well-being of older Japanese adults, particularly in a post-COVID-19 pandemic context [12,13,14]. For example, a longitudinal study of older Japanese adults revealed that those with decreased social participation due to the pandemic had increased risk of developing symptoms of depression [14], while another report linked COVID-19 countermeasures with increased frailty among the same demographic [15]. Within this context, volunteering presents a potential avenue to enhance the quality of life and social engagement of older adults, yet the specific impact of volunteer activities on the well-being of this population, particularly in a post-pandemic context, remains somewhat under-explored. Understanding the associations between volunteering and health outcomes in older Japanese adults can provide valuable insights into how volunteering might serve as an effective public health intervention in promoting healthier aging.

In a previous study, which focused primarily on the sleep quality and duration of older adults with hypertension, we found that volunteers experienced improved sleep quality (including less nocturnal awakenings) and lower levels of depression than non-volunteers [16]. Building upon this work, the aim of the current study was to investigate the differences in demographic, health, and lifestyle characteristics between volunteers and non-volunteers among a cohort of older Japanese adults. By utilizing an online survey conducted during the COVID-19 pandemic, the study sought to explore the associations between a range of key physical and mental health indicators, including sleep, dysphagia risk, depression, loneliness, and social engagement, to explore the health, well-being, and lifestyle characteristics of active volunteers. The timing of this research, during the pandemic, adds another layer of importance, as the restrictions on social interactions imposed by COVID-19 have exacerbated issues, such as loneliness and mental health challenges, particularly among older adults. In response to concerns about increased social isolation among the Japanese population, in 2024, the Cabinet Office established the Headquarters for Advancement of Measures to Address Loneliness and Isolation [17]. The aim of this initiative is to focus on implementing necessary measures to prevent loneliness and social isolation by promoting continued social connections [17]. Additionally, the World Health Organization (WHO) has recognized loneliness and social isolation as global public health concerns and has established the Commission on Social Connection to address these issues [18]. We hypothesize that engagement in volunteer activities, perhaps within a framework such as social prescribing, could help to tackle loneliness and social isolation by encouraging participants to be more active and encourage social connection in meaningful roles within the community. Furthermore, identifying the health and lifestyle characteristics typical of volunteers could help healthcare providers identify those individuals for whom engagement with volunteering could be particularly helpful.

Therefore, the purpose of the current study was to explore the associations between volunteering and various physical and mental health measures among a cohort of older Japanese adults. The study contributes new insights into how volunteering can support healthier aging in the specific post-pandemic context, incorporating a broad range of health indicators and aligning with Japanese public health initiatives. Our findings add to the growing body of evidence that affirms the role of volunteering as a critical component in promoting healthier aging and provides implications for public health strategies, suggesting that promoting volunteer opportunities for older adults could enhance their well-being and mitigate some of the challenges associated with aging, such as loneliness, social isolation, and mental health decline.

## 2. Materials and Methods

### 2.1. Participants

The survey used in this study was conducted from 1 to 20 June 2021 using an online questionnaire that was deemed most suitable, as it was carried out during the COVID-19 pandemic. Participants were recruited through Rakuten Insight (Rakuten Insight Global, Inc., Tokyo, Japan), an academic research internet survey platform with 2.2 million registered users representing a broad and diverse demographic across various of ages and regions. Rakuten Insight offers efficient access to a representative study population while ensuring data quality, security, and compliance. The platform’s extensive reach allows for the collection of data that closely mirrors Japan’s general population. The inclusion criteria for participants were: (1) aged 65 years or older, (2) living independently, and (3) capable of independently completing the questionnaire. The exclusion criterion was a diagnosis of dementia. The participants were divided into two groups, volunteer group and non-volunteer group, based on their involvement in volunteering. Participants who indicated “volunteering” in the Daily activities section of the survey were classified as the “volunteer group”, while those who did not were classified as the “non-volunteer group”.

As in our previous publication [19], in the a priori power analysis, the sample size was determined factoring in the 53.4% internet usage rate among those aged 65+ in 2021 [20]. Using G*Power (G*Power for Windows 3.1.9.7, RRID:SCR_013726, Dusseldorf, Germany [21]), the sample size was calculated to achieve 95% power, with an alpha (α) error of 5% and a medium effect size, resulting in a minimum required sample size of *N* = 462. Therefore, the final sample size of 500 in this study provides sufficient statistical power to detect meaningful effects.

### 2.2. Questionnaire

#### 2.2.1. Demographic Characteristics

The demographic characteristics assessed in the survey included: (1) sex and age, (2) height, weight, and BMI, (3) occupation, (4) educational background, (5) daily activities (e.g., housework, volunteering, hobbies), (6) frequency of outings per week (the number of times they leave the home) before and during the COVID-19 pandemic, (7) family and marital status, (8) discussion partners for important matters, (9) current diseases under treatment, and (10) smoking and drinking habits.

#### 2.2.2. Assessment

Participants’ physical and mental health status was comprehensively assessed using the Japanese versions of the following six scales: (1) the Pittsburgh Sleep Quality Index (PSQI), (2) the Dysphagia Risk Assessment for Community-Dwelling Elderly (DRACE) instrument, (3) the Geriatric Depression Scale 15 (GDS-15), (4) the University of California, Los Angeles Loneliness Scale 3 (UCLA LS3), (5) Kihon Checklist (KCL), (6) the Short Form-8 (SF-8) health survey, and (7) the Physical Activity Scale for the Elderly (PASE).

The PSQI assesses sleep disorders in older adults based on sleep patterns over the last month. Each item is scored from 0 to 3, resulting in a total score ranging from 0 to 21. Higher scores indicate more severe sleep disorders, with a cutoff point of 5/6 [22,23]. The Japanese version of this scale has been shown to have high overall reliability (Cronbach’s alpha = 0.77) [22].

The DRACE, which consists of 12 items, evaluates dysphagia risk in community-dwelling older adults based on swallowing function over the last year [24]. Each item is scored from 0 to 2, with a total score ranging from 0 to 24. Scores of 5 or higher indicate a high risk of dysphagia. The DRACE has demonstrated satisfactory reliability with a Cronbach’s alpha coefficient of 0.88 [24].

The GDS-15 assesses depression in older adults using 15 questions. Each item is scored from 0 to 1, with a total score ranging from 0 to 15. Scores of 5 to 9 indicate a tendency toward depression, while scores of 10 or higher indicate depression. The Japanese version exhibited a high degree of internal consistency, as evidenced by a Cronbach’s alpha reliability coefficient of 0.83 [25].

The UCLA LS, now in its third version (UCLA-LS3), assesses loneliness in adults and has demonstrated reliability and validity [26]. The Japanese version evaluates loneliness through 20 questions, with each item scored from 1 to 4, resulting in a total score ranging from 20 to 80. Higher scores indicate greater loneliness [27]. The validity and reliability of the Japanese version of the UCLA LS has been demonstrated with a Cronbach alpha of 0.92 [27].

The KCL, developed by the Japanese Ministry of Health, Labour and Welfare (MHLW), is used to screen for frailty as a means of assessing the level of care required by the individual [28]. It assesses activities of daily living (ADL), physical function, nutrition, swallowing, being housebound, cognitive function, and depression through 25 questions, each scored from 0 to 1, with a total score ranging from 0 to 25. Higher scores indicate greater frailty and therefore a higher level of care need. Previous studies have validated the KCL as a measure of frailty [29].

The SF-8 measures health-related quality of life across 8 domains: physical functioning, role physical, bodily pain, general health, vitality, social functioning, role emotional, and mental health [30]. The SF-8 renders both a physical component score (PCS) and mental component score (MCS). The Japanese version of the SF-8 has been shown to be particularly effective at identifying individuals with lower QOL [31].

The PASE is a survey instrument used to assess physical activity levels in older adults. It evaluates the frequency, duration, and intensity of activities, including leisure (walking, exercise), household (housework, gardening), and work-related activities. Respondents report their weekly activity, which is used to calculate a composite score, with higher PASE scores indicating higher levels of physical activity [32]. The Japanese version of this scale has demonstrated acceptable validity and reliability with an intraclass correlation coefficient of 0.65 [32].

### 2.3. Statistical Analysis

The chi-squared and the Mann–Whitney U tests were used for between-group comparisons. To explore the associations between the health and lifestyle characteristics of active volunteers and non-volunteers, logistic regression analysis using the forced entry method was conducted for items that showed significant differences between the two groups, with “volunteering” set as the outcome variable. The significance level was set at *p* < 0.05 or *p* < 0.01. SPSS version 29.0.1.0 (IBM Corporation, Armonk, NY, USA) was used for the analysis.

### 2.4. Ethics

This study was conducted in accordance with guidelines of the Declaration of Helsinki. Informed consent was obtained from all participants, as outlined by Rakuten Insight (https://insight.rakuten.co.jp/company/privacy.html (accessed on 17 February 2023)). This study was approved by the by the ethics committee of the Institute of Medicine, University of Tsukuba (approval 1623-5).

## 3. Results

### 3.1. Participant Demographics and Characteristics

A total of 500 older adults (250 females and 250 males) participated in the survey, with 64 individuals allocated to the volunteer group and 436 to the non-volunteer group. The results of the survey are given in Table 1. The average age of the entire cohort was approximately 73.57 years, with no significant age difference between volunteers and non-volunteers. Gender distribution showed an even split between males and females in both groups. The average BMI for the cohort was 22.60, with no significant differences between volunteers and non-volunteers. The majority of individuals did not live alone, but rather lived with a partner or family (child and/or grandchild), and no significant differences in living arrangements were observed between the groups. Around 28% of the cohort were engaged in part-time or full-time work, again with no significant differences between the groups. 

There were significant differences between the two groups in terms of housework and hobbies and lessons, with reduced activities among the non-volunteer group. The volunteer group was more likely to engage in housework (χ^2^(1) = 5.102, *p* = 0.024) and hobbies/lessons (χ^2^(1) = 15.328, *p* = < 0.001) than the non-volunteer group. The number of days per week that the participants went on outings was not significantly different between the two groups before and during the COVID-19 pandemic period. However, before COVID-19, participants who went out one day or more per week were more prevalent in the volunteer group than in the non-volunteer group (χ^2^(1) = 4.296, *p* = 0.038), but this difference was not observed during COVID-19. Other characteristics did not show significant differences between the groups.

Table 2 shows the diseases under treatment among the study cohort. They reported various diseases under treatment, with notable conditions including hypertension (32.0%), diabetes (10.8%), lower back pain (10.6%), and urological disease (8.6%). However, differences between volunteers and non-volunteers were statistically insignificant for most of the conditions, except for a higher prevalence of cataracts under treatment among volunteers (*p* = 0.014).

### 3.2. Psychosomatic Status

The GDS-15 and the UCLA LS3 showed significant differences between the two groups. The volunteer group had lower total GDS-15 scores than the non-volunteer group (2.69 ± 2.67 vs. 3.61 ± 3.15, *p* < 0.024) and were significantly less likely to exhibit depressive tendencies (*p* < 0.021). Additionally, the volunteer group had significantly lower scores on the Japanese version of the UCLA LS3, indicating less loneliness compared to the non-volunteer group (35.39 ± 11.31 vs. 40.97 ± 9.91, *p* < 0.001). There were no significant differences between the two groups in PSQI, DRACE, KCL, or SF-8 scores. As a result, housework, hobbies, going out one day or more per week, the GDS-15, and the UCLA LS3 were included in the logistic regression analysis. Because housework is often performed by women in Japan, gender was added as a control variable to account for its potential influence on the relationship between housework and volunteering. The analysis showed significant differences for housework (OR = 1.726, CI = 0.827–3.605), hobbies/lessons (OR = 2.667, CI = 1.491–4.722), and the UCLA LS3 (OR = 0.950, CI = 0.919–0.983) (Table 3).

## 4. Discussion

The purpose of this study was to examine the differences in various demographic, health, and lifestyle characteristics between volunteers and non-volunteers in a cohort of older Japanese adults in an effort to explore the associations between volunteering and possible health and social benefits. The findings reveal several trends and associations. The demographic analysis showed no significant differences in age or gender distribution between volunteers and non-volunteers, indicating that these factors do not significantly influence the likelihood of volunteering among older Japanese adults. Kobayashi and colleagues also found that gender did not have a significant main effect on volunteer participation among a Japanese cohort [33]. On the other hand, a study by Tomioka et al. showed that Japanese male older adults were significantly (*p* < 0.001) more likely to be engaged in volunteer groups and that volunteerism had a favorable impact on their instrumental activities of daily living [34]; however, in our survey we did not specify “volunteer group”, and thus the volunteering captured in our data likely encompassed a broader range of activities. A review by Musick et al. revealed that women tend to participate in volunteer activities more frequently than men, and the authors reasoned that women generally exhibit greater compassion than men, which often leads to their increased involvement in nurturing and caregiving roles [35]; however, they remarked that the difference is not great [35]. The older age of the participants of the current study may well account for the lack of difference between the genders in our data.

In a previous study of older Japanese adult volunteers, Lee and colleagues found that women living with a partner were less likely to participate in volunteering compared to those living alone [36]. Similarly, Principi and colleagues examined the association between the recourses and motivations of older adult volunteers in Europe and discovered that lower levels of human and social capital, including being widowed, divorced, or single, were linked to a greater likelihood of volunteering [37]. However, in the current study, the living arrangements of the participants, whether living alone, with a partner, or with family, did not differ significantly between the two groups. This suggests that volunteering behavior in older Japanese adults is largely unrelated to their living situation.

Among our older adult cohort, various diseases were reported to be under treatment, with hypertension, diabetes mellitus, and lower back pain showing the highest prevalence for both volunteers and non-volunteers without any significant differences. In fact, there were no major statistical differences in diseases under treatment between volunteers and non-volunteers, except for cataracts. Among a large Canadian cohort of older adults, the presence of visual impairment and glaucoma was found to be associated with reduced social participation [38]. This might explain the higher prevalence of volunteers receiving treatment for cataracts in comparison to those among the non-volunteers, who may have a similar frequency of cataracts, but may not be seeking treatment. Good visual health is an essential factor that enables older adults to remain actively engaged in social activities, as it supports better mobility, independence, and confidence in navigating various social settings. Visual impairments play a critical role in fall events among older adults and can be isolating, limiting the ability to interact comfortably in both structured volunteer roles and casual social gatherings [39]. Therefore, maintaining or improving visual health through treatments such as cataract surgery may contribute to increased social participation among older adults [40].

Although there were no significant differences in sleep quality (PSQI) or pulmonary aspiration risk (DRACE) between the groups, the overall physical and mental component summary scores (PCS and MCS) of the SF-8 were slightly higher for volunteers, though not significantly. This trend suggests a potential, albeit small, benefit of volunteering on overall perceived health and quality of life. Other studies also point to the impact of volunteering on perceived health status [41,42,43]. A study of older Taiwanese adults (that utilized the SF-12) found volunteering to be significantly linked to improved subjective physical health, higher self-rated health, and greater self-rated happiness [41]. Self-rated health measurement, widely used in epidemiological, clinical, and social research, is an important predictor of actual health status and mortality [44], and recent work has pointed to its strong biological basis being associated with various biomarkers [45]. In light of these findings, the positive influence of volunteering on perceived health and well-being should not be underestimated. Given the growing body of research supporting these associations, further exploration into the nuances of volunteering’s impact on subjective health could yield valuable insights for promoting healthier aging populations.

While many of the physical health indicators were similar between volunteers and non-volunteers in the current study, there were some significant differences in mental health indicators. Volunteers in our study had significantly lower Geriatric Depression Scale (GDS) scores, indicating lower levels of depression compared to non-volunteers. This aligns with previous research suggesting that engaging in volunteer activities can have protective effects against depression and positively affect the well-being of older adults [1,2,46,47]. Our finding is supported by another study among older Japanese adults, the findings of which demonstrated that individuals who engaged in volunteer activities at least once a month had a “28% lower risk of developing depressive symptoms” [48]. Evidence suggests that various psychosocial factors contribute to the protective effect against the development of depressive symptoms among volunteers. In older adults, volunteering has been shown to increase the sense of self-esteem and purpose [4,5,40,49] and increased social networks and social support [50,51,52].

In the current study, volunteers also reported significantly lower levels of loneliness. Loneliness in old age, compounded by the social distancing and isolation that were imposed during the COVID-19 pandemic, can have far-reaching negative effects, impacting not only emotional well-being but also physical and mental health. Prolonged feelings of loneliness are associated with an increased risk of chronic illnesses, such as cardiovascular disease [53,54] and weakened immune function [55,56,57]. Mentally, loneliness can contribute to the onset or worsening of conditions like depression and anxiety [58,59,60]. Additionally, it has been linked to cognitive decline and may actually accelerate the progression of neurodegenerative diseases such as Alzheimer’s [61,62,63]. The adverse consequences of loneliness in older adults emphasize the importance of social connections and support networks in promoting a healthier and longer life. The results of our study, along with others [64,65,66], suggest that volunteering could be a powerful means of addressing this pressing issue of loneliness among older adults.

The volunteers in our study appeared to be more engaged in physical and social activities compared to non-volunteers. A significantly higher percentage of volunteers participated in hobbies and/or lessons, and (prior to the COVID-19 pandemic) they were more likely to go on outings. While it is not possible to show from the current data whether volunteering promotes a more active lifestyle or vice versa, this observation indicates that volunteering forms part of a holistic, healthy, active, and engaged lifestyle. Another study among older Japanese adults found that participants involved in volunteer activities, sports, and hobby groups were more likely to adopt healthy lifestyle behaviors compared to those who did not participate in such groups [67]. Furthermore, social activities, like hobbies and lessons, likely further contribute to the enhanced mental health and lower loneliness observed among volunteers in our study. Unfortunately, the COVID-19 pandemic had a noticeable impact on the frequency of outings for both volunteers and non-volunteers, with a general reduction observed during the pandemic. However, the decline was not statistically significant between the groups, suggesting that while the pandemic affected social activities, the inherent benefits associated with volunteering persisted to some extent.

In line with the successful aging model [68] and the productive aging theory [69], the findings of this study further emphasize the importance of volunteering as a pathway to healthier aging. According to the successful aging model, put forward by Rowe and Kahn, active engagement with life is one of the three core components of successful aging, alongside low probability of disease and high functional capacity [68]. In their seminal work, Rowe and Kahn discuss volunteering as one of the major productive activities that older-adults can engage in [68]. Our results, showing reduced loneliness and depressive symptoms among volunteers, suggest that volunteering may promote both social engagement and psychological well-being, two essential factors for maintaining an active and fulfilling life in older adulthood. Additionally, Butler and Schechter’s productive aging theory highlights the value of older adults continuing to contribute meaningfully to society, especially through volunteer activities, which not only benefits their own mental health and sense of purpose but also adds social value [69]. Volunteering, like caregiving for a loved one [70], enables older adults to maintain a productive role within their community, which supports both their psychological resilience and broader public health goals by reducing the risk of social isolation.

The findings of our study suggest the potential of volunteer activities as an effective, low-cost intervention to improve mental health and encourage social engagement among older adults. Volunteering not only offers a sense of purpose and fulfillment but also creates opportunities for meaningful social connections, which are crucial for combating loneliness and isolation in this population. Given these substantial benefits, developing programs that actively encourage and facilitate volunteerism could be instrumental in promoting healthier and more engaged communities. Indeed, a number of studies have utilized volunteering-based interventions to study the impact of volunteering on the well-being of older adults [71,72,73]. An investigation by Sato and colleagues into community-based programs to promote social engagement showed an 11% reduction in the likelihood of frailty among older Japanese adults [74], which has large implications for reducing healthcare costs. Thus, healthcare providers and policymakers should view volunteering not merely as a recreational activity, but as a strategic tool to reduce healthcare burdens (including financial concerns) and contribute to a more vibrant and resilient aging population. By integrating volunteer opportunities into public health strategies, we can better address the complex needs of older adults, ensuring that they remain active participants in society while also improving their overall quality of life.

In recent years, social prescribing has gained attention as an approach to addressing the social, emotional, and practical needs of older adults [75,76,77]. Social prescribing involves healthcare professionals referring patients to non-clinical services within their community, including volunteer activities, to improve well-being and overall health [75,76,77]. Volunteering, which is emerging as an important component of social prescribing, offers older adults structured opportunities to engage with their communities, enhancing their social networks and reducing isolation—two factors strongly linked to better health outcomes [78,79,80]. Some recent studies have described such social prescribing interventions in Japanese settings [81,82,83]. In Japan, where strong social ties have historically been a key element of well-being, social prescribing—especially through volunteer activities—could help combat rising rates of loneliness and isolation among older adults. Furthermore, the findings of our study could possibly help to identify those older Japanese adults for whom volunteering could be a particularly useful social prescription. Given the association between volunteering with reduced loneliness and depressive symptoms revealed in the current study, incorporating volunteering into social prescribing could become a useful strategy for promoting healthier, more active aging populations and reducing the burden on healthcare systems, aligning with the country’s broader goals of promoting preventive care and social engagement.

### Limitations

This study has several limitations. Firstly, it must be mentioned that the cross-sectional design precludes any causal inferences, i.e., it is impossible to infer from our data whether volunteering improves/maintains health status or whether healthier individuals are more likely to volunteer. Longitudinal research and evidence from systematic reviews exploring this question point to the bidirectional, reciprocal relationship between health and volunteering [84,85,86]. While further longitudinal research would be required to explore the direction of causality within our study population, our findings add to the growing body of evidence supporting the positive association between volunteering and health. Secondly, the sample size, while adequate for the study, may not fully represent the broader population of older Japanese adults. In particular, the proportion of volunteers was quite small, which may have been an effect of the COVID-19 pandemic, which limited the number of volunteer opportunities. Additionally, self-reported measures can be subject to recall bias and may not accurately capture all aspects of physical activity or health status. Furthermore, the survey lacked detail about the type of volunteer activity and the amount of time involved in volunteering, which would have helped to bring more granularity and nuance to the findings. Despite these limitations, our study of 500 community-dwelling older Japanese adults offers further insights into the associations between volunteering and various aspects of health and well-being from a time during the COVID-19 pandemic when they were at particular risk of loneliness, isolation, and ill health.

## 5. Conclusions

In conclusion, this study highlights the valuable role that volunteering could play in enhancing or maintaining the mental health, social engagement, and overall well-being of older adults. While no significant differences were found in physical health outcomes, mental health indicators such as lower depression and loneliness were significant between volunteers and non-volunteers. These findings, supported by previous research, suggest that volunteer activities could be a crucial component of public health strategies, such as social prescribing, aimed at promoting healthier aging. Future research should explore volunteering at a more granular level, investigate its longitudinal effects, and identify potential mechanisms through which volunteering influences health outcomes in older adults. Encouraging older adults to engage in volunteering as a means of social connection may not only improve their quality of life but also help mitigate healthcare burdens by reducing risks associated with mental health issues and social isolation, which is becoming increasingly important in our aging societies.

## Figures and Tables

**Table 1:** Participant demographic characteristics and survey results.

		Entire Cohort (*n* = 500)	Volunteer (*n* = 64)	Non-Volunteer (*n* = 436)	*p*	χ^2^
Age (years)		73.57 ± 8.93	74.5 ± 4.80	73.43 ± 5.63	0.083	
Gender	Male	250 (50%)	26 (40.3%)	224 (51.4%)	0.070	2.580
	Female	250 (50%)	38 (59.7%)	212 (48.6%)		
BMI		22.60 ± 3.29	22.63 ± 2.72	22.59 ± 3.37	0.834	
Family:						
Living alone	Yes	91 (18.2%)	10 (15.6%)	81 (18.6%)	0.726	0.212
	No	384 (76.8%)	49 (76.6%)	335 (76.8%)		
With child/grandchild	Yes	15 (3%)	1 (1.6%)	14 (3.2%)	0.706	0.471
	No	460 (92%)	58 (90.6%)	402 (92.2%)		
With partner	Yes	258 (51.6%)	34 (53.1%)	224 (51.4%)	0.676	0.298
	No	217 (43.4%)	25 (39%)	192 (44%)		
Occupation	Yes	141 (28.2%)	17 (26.6%)	124 (28.4%)	0.882	0.097
	No	359 (71.8%)	47 (73.4%)	312 (71.6%)		
Daily activities:						
Housework	Yes	328 (65.6%)	50 (78.1%)	278 (63.8%)	0.024	5.102
	No	172 (34.4%)	14 (21.9%)	158 (36.2%)		
Hobbies/lessons	Yes	175 (35%)	42 (65.6%)	173 (39.7%)	<0.001	15.328
	No	285 (56.9%)	22 (34.4%)	263 (60.3%)		
Frequency of outings/week:						
Before COVID-19		4.26 ± 2.11	4.60 ± 1.74	4.21 ± 2.16	0.283	
During COVID-19		3.47 ± 2.36	3.67 ± 2.10	3.44 ± 2.40	0.439	
Outing days/week:						
Before COVID-19	Yes (≥1/week)	459 (91.8%)	63 (98.4%)	396 (90.8%)	0.047	4.296
	No (rarely)	41 (8.2%)	1 (1.6%)	40 (9.2%)		
During COVID-19	Yes (≥1/week)	418 (83.6%)	57 (1.6%)	361 (82.8%)	0.277	1.597
	No (rarely)	82 (16.4%)	7 (1.6%)	75 (17.2%)		
Diseases under treatment		1.18 ± 1.34	1.43 ± 1.48	1.15 ± 1.31	0.129	
GDS score		3.49 ± 3.11	2.69 ± 2.67	3.61 ± 3.15	0.024	
Depression (GDS ≥ 5)	Yes	156 (31.2%)	12 (18.8%)	144 (33%)	0.021	5.300
	No	344 (68.8%)	52 (81.2%)	292 (67%)		
PSQI (mean ± SD)		4.79 ± 2.50	5.00 ± 2.46	4.76 ± 2.51	0.432	
Sleep disorder (PSQI ≥ 6)	Yes	165 (33%)	21 (32.8%)	144 (33%)	1.00	0.001
	No	335 (67%)	43 (62.2%)	292 (67%)		
SF-8 PCS		49.55 ± 6.03	50.04 ± 5.98	49.48 ± 6.04	0.449	
SF-8 MCS (mean ± SD)		52.27 ± 5.09	52.02 ± 5.63	52.30 ± 5.01	0.577	
DRACE score		2.34 ± 3.07	2.23 ± 2.15	2.36 ± 3.19	0.392	
Pulmonary aspiration risk, DRACE ≥ 4	Yes	125 (25%)	16 (25%)	109 (25%)	1.00	0.000
	No	375 (75%)	48 (75%)	327 (75%)		
UCLA-LS3		40.25 ± 10.26	35.39 ± 11.31	40.97 ± 9.91	<0.001	
KCL		5.71 ± 3.29	5.5 ± 2.9	5.74 ± 3.34	0.902	
Frailty KCL ≥5	Robust	230 (46%)	30 (46.9%)	200 (45.9%)	0.894	0.023
	Prefrail/frail	270 (54%)	34 (53.1%)	236 (54.1%)		
PASE		95.99 ± 53.58	99.39 ± 45.13	95.49 ± 54.74	0.216	

PSQI, Pittsburgh Sleep Quality Index; DRACE, Dysphagia Risk Assessment for Community-Dwelling Elderly; GDS-15, Geriatric Depression Scale 15; UCLA LS3, University of California, Los Angeles Loneliness Scale 3; KCL, Kihon Checklist; SF-8, Short Form 8 health survey; PASE, Physical Activity Scale for the Elderly. Mean ± SD, χ^2^ test, Mann–Whitney U test.

**Table 2:** Diseases under treatment among the study cohort.

	Entire Cohort (Total *n* = 500) *n* (%)	Volunteer (Total *n* = 64) *n* (%)	Non-Volunteer (Total *n* = 436) *n* (%)	*p*	χ^2^
Hypertension	160 (32.0)	23 (35.9)	137 (31.4)	0.278	0.523
Heart disease	34 (6.8)	7 (10.9)	27 (6.2)	0.180	1.983
Arteriosclerosis	8 (1.6)	2 (3.1)	6 (1.4)	0.273	1.084
Cerebrovascular disease	7 (1.4)	0 (0)	7 (1.6)	0.603	1.042
Gastrointestinal disease	20 (4.0)	1(1.6)	19(4.4)	0.494	1.136
Pneumonia	8 (1.6)	2 (3.1)	6 (1.4)	0.273	1.084
Asthma	14 (2.8)	3 (4.7)	11 (2.5)	0.404	0.961
Chronic obstructive pulmonary disease	1 (0.2)	0 (0)	1 (0.2)	1.000	0.147
Hay fever/allergy	38 (7.6)	4 (6.3)	34 (7.8)	0.804	0.190
Urological disease	43 (8.6)	3 (4.7)	40 (9.2)	0.338	1.429
Liver disease	2 (0.4)	1 (1.6)	1 (0.2)	0.240	2.490
Neurological disease	3 (0.6)	1 (1.6)	2 (0.5)	0.338	1.140
Lower back pain	53 (10.6)	11 (17.2)	42 (9.6)	0.080	3.361
Mental illness	1 (0.2)	0 (0)	1 (0.2)	1.000	0.147
Neurosis	5 (1.0)	0 (0)	5 (1.1)	1.000	0.741
Autonomic nervous system disorder	2 (0.4)	1 (1.6)	1 (0.2)	0.240	2.490
Cold	1 (0.2)	0 (0)	1 (0.2)	1.000	0.147
Dermatological disease	21 (4.2)	3 (4.7)	18 (4.1)	1.000	0.002
Cataract	27 (5.4)	8 (12.5)	19 (4.4)	0.014	7.243
Sleep disorder	10 (2.0)	0 (0)	10 (2.3)	0.624	1.498
Diabetes mellitus	54 (10.8)	9 (14.1)	45 (10.3)	0.387	0.811

**Table 3:** Logistic regression analysis.

	Odds Ratio	95%CI	*p*
Gender	1.283	0.666–2.470	0.456
Housework	1.726	0.827–3.605	0.146
Hobbies/lessons	2.667	1.491–4.722	<0.001
Outing days/week (before COVID-19)	3.907	0.509–29.985	0.190
GDS-15 score	0.955	0.887–1.117	0.937
UCLA LS3	0.950	0.919–0.983	0.003

95% CI, 95% confidence interval; GDS-15, Geriatric Depression Scale 15; UCLA LS3, University of California, Los Angeles Loneliness Scale 3.

## Data Availability

The original contributions presented in the study are included in the article. Further inquiries can be directed to the corresponding author.
